# 5-(4-Chloro­phen­yl)-1-cyclo­propyl-2-(2-fluoro­phen­yl)-3-phenyl­pentane-1,5-dione

**DOI:** 10.1107/S1600536813001074

**Published:** 2013-01-19

**Authors:** Thothadri Srinivasan, Govindaraj Senthilkumar, Haridoss Manikandan, Kaliaperumal Neelakandan, Devadasan Velmurugan

**Affiliations:** a Centre of Advanced Study in Crystallography and Biophysics, University of Madras, Guindy Campus, Chennai 600 025, India; b Department of Chemistry, Annamalai University, Annamalainagar 608 002, Tamilnadu, India

## Abstract

In the title compound, C_26_H_22_ClFO_2_, the cyclo­propane ring makes dihedral angles of 45.7 (2), 49.0 (2) and 65.2 (2)° with the fluoro-substituted phenyl ring, the benzene ring and the chloro-substituted phenyl ring, respectively. The F and Cl atoms deviate by 0.0307 (11) and 0.0652 (6) Å, respectively, from the planes of the phenyl rings to which they are attached. In the crystal, mol­ecules are linked by C—H⋯F hydrogen bonds, forming chains along the *b* axis.

## Related literature

For the uses and biological importance of diketones, see: Bennett *et al.* (1999 ▶); Sato *et al.* (2008 ▶). For a related structure, see: Li *et al.* (2008 ▶).
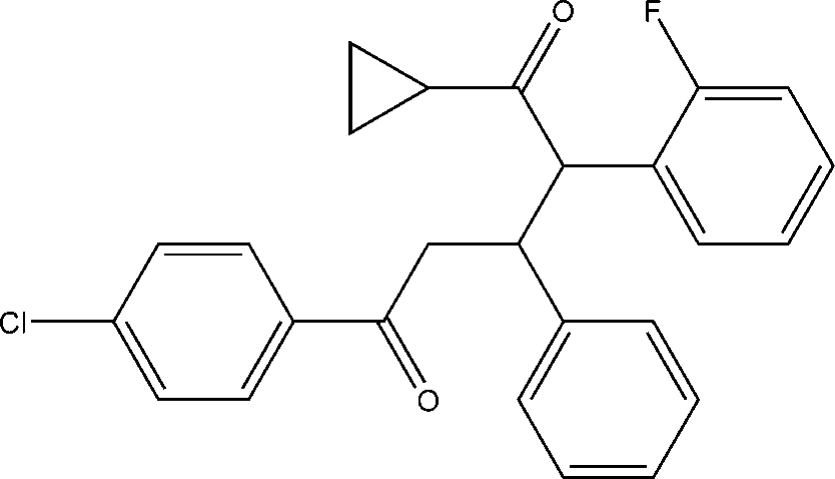


## Experimental

### 

#### Crystal data

C_26_H_22_ClFO_2_
*M* *_r_* = 420.89
Monoclinic, 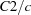

*a* = 43.0465 (15) Å
*b* = 5.7257 (2) Å
*c* = 18.2828 (6) Å
β = 109.103 (2)°
*V* = 4258.0 (3) Å^3^
*Z* = 8
Mo *K*α radiation
μ = 0.21 mm^−1^
*T* = 293 K
0.30 × 0.25 × 0.20 mm

#### Data collection

Bruker SMART APEXII area-detector diffractometer
Absorption correction: multi-scan (*SADABS*; Bruker, 2008 ▶) *T*_min_ = 0.940, *T*_max_ = 0.960
20606 measured reflections
5308 independent reflections
3857 reflections with *I* > 2σ(*I*)
*R*_int_ = 0.026

#### Refinement

*R*[*F* ^2^ > 2σ(*F* ^2^)] = 0.045
*wR*(*F* ^2^) = 0.146
*S* = 1.02
5308 reflections
271 parameters
H-atom parameters constrained
Δρ_max_ = 0.28 e Å^−3^
Δρ_min_ = −0.41 e Å^−3^

### 

Data collection: *APEX2* (Bruker, 2008 ▶); cell refinement: *SAINT* (Bruker, 2008 ▶); data reduction: *SAINT*; program(s) used to solve structure: *SHELXS97* (Sheldrick, 2008 ▶); program(s) used to refine structure: *SHELXL97* (Sheldrick, 2008 ▶); molecular graphics: *ORTEP-3 for Windows* (Farrugia, 2012 ▶); software used to prepare material for publication: *SHELXL97* and *PLATON* (Spek, 2009 ▶).

## Supplementary Material

Crystal structure: contains datablock(s) global, I. DOI: 10.1107/S1600536813001074/su2549sup1.cif

Structure factors: contains datablock(s) I. DOI: 10.1107/S1600536813001074/su2549Isup2.hkl

Supplementary material file. DOI: 10.1107/S1600536813001074/su2549Isup3.cml

Additional supplementary materials:  crystallographic information; 3D view; checkCIF report

## Figures and Tables

**Table 1:** Hydrogen-bond geometry (Å, °)

*D*—H⋯*A*	*D*—H	H⋯*A*	*D*⋯*A*	*D*—H⋯*A*
C12—H12⋯F1^i^	0.98	2.54	3.433 (2)	151

Symmetry code: (i) 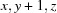.

**Fig. 1.**
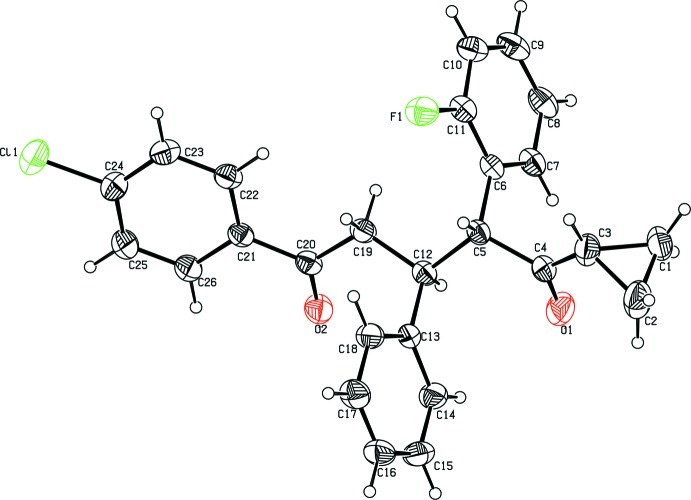
The molecular structure of the title molecule, with atom labelling. Displacement ellipsoids are drawn at the 30% probability level.

**Fig. 2.**
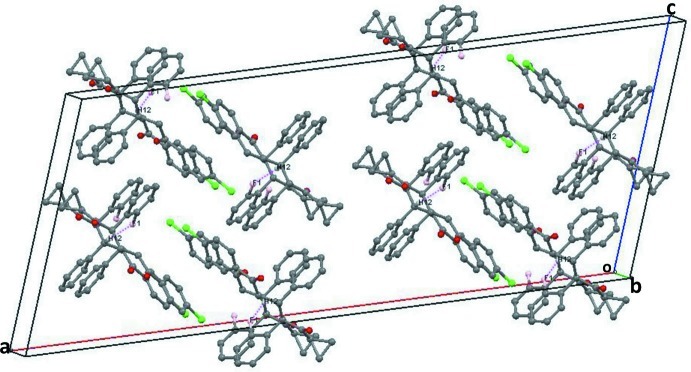
The crystal packing of the title compound viewed along the b axis. H-atoms not involved in C-H···F hydrogen bonds (dashed lines) have been excluded for clarity.

## supplementary crystallographic information

### Comment

Diketones are popular in organic synthesis for their applications in biology and medicine. They are known to exhibit antioxidant, antitumour and antibacterial activities (Bennett *et al.*, 1999). They are also key intermediates in the preparation of various heterocyclic compounds (Sato *et al.*, 2008). We report herein on the synthesis and crystal structure of one the title diketone.

In the title compound, Fig.1, the cyclopropane ring (C1-C3) makes a dihedral angle of 45.7 (2)° with the fluoro substituted phenyl ring (C6-C11). It makes a dihedral angle of 49.0 (2)° with the unsubstituted phenyl ring (C13-C18) and a dihedral angle of 65.2 (2)° with the chloro substituted phenyl ring (C21-C26). The fluorine atom, F1, deviates by 0.0307 (11) Å from the phenyl ring to which it is attached.

The dihedral angle between the unsubstituted phenyl ring and the fluoro substituted phenyl ring is 3.65 (8)° and the dihedral angle between the unsubstituted phenyl ring and the chloro substituted phenyl ring is 71.73 (8)°. The dihedral angle between the fluoro substituted phenyl ring and the chloro substituted phenyl ring is 71.15 (8)°. The chloro atom, Cl1, deviates by 0.0652 (6) Å from the phenyl ring to which it is attached.

In the crystal, C–H···F hydrogen bonds link the molecules to form chains along the b axis (Table 1 and Fig. 2).

### Experimental

A mixture of 4-chloroacetophenone(0.01mole), benzaldehyde (0.01 mole), cyclopropyl 2-fluorobenzyl ketone (0.01 mole) and sodium hydroxide solution (10 ml, 10%) in ethanol (50 ml) was stirred for 3 hrs at room temperature. The solid that separated was filtered and washed with distilled water. The product was recrystallised from ethanol [Yield = 95%, M.p. = 405 - 407 K] giving block-like colourless crystals of the title compound.

### Refinement

The hydrogen atoms were placed in calculated positions and treated as riding atoms: C—H = 0.93 - 1.08 Å with *U*_iso_(H) = 1.5*U*_eq_(C) for methyl H atoms and = 1.2*U*_eq_(C) for other H atoms.

### Crystal data

**Table:**

C_26_H_22_ClFO_2_	*F*(000) = 1760
*M**_r_* = 420.89	*D*_x_ = 1.313 Mg m^−^^3^
Monoclinic, *C*2/*c*	Mo *K*α radiation, λ = 0.71073 Å
Hall symbol: -C 2yc	Cell parameters from 5308 reflections
*a* = 43.0465 (15) Å	θ = 2.0–28.3°
*b* = 5.7257 (2) Å	µ = 0.21 mm^−^^1^
*c* = 18.2828 (6) Å	*T* = 293 K
β = 109.103 (2)°	Block, colourless
*V* = 4258.0 (3) Å^3^	0.30 × 0.25 × 0.20 mm
*Z* = 8	

### Data collection

**Table:**

Bruker SMART APEXII area-detector diffractometer	5308 independent reflections
Radiation source: fine-focus sealed tube	3857 reflections with *I* > 2σ(*I*)
Graphite monochromator	*R*_int_ = 0.026
ω and φ scans	θ_max_ = 28.3°, θ_min_ = 2.0°
Absorption correction: multi-scan (*SADABS*; Bruker, 2008)	*h* = −56→55
*T*_min_ = 0.940, *T*_max_ = 0.960	*k* = −7→7
20606 measured reflections	*l* = −24→24

### Refinement

**Table:**

Refinement on *F*^2^	Primary atom site location: structure-invariant direct methods
Least-squares matrix: full	Secondary atom site location: difference Fourier map
*R*[*F*^2^ > 2σ(*F*^2^)] = 0.045	Hydrogen site location: inferred from neighbouring sites
*wR*(*F*^2^) = 0.146	H-atom parameters constrained
*S* = 1.02	*w* = 1/[σ^2^(*F*_o_^2^) + (0.0784*P*)^2^ + 1.3969*P*] where *P* = (*F*_o_^2^ + 2*F*_c_^2^)/3
5308 reflections	(Δ/σ)_max_ = 0.001
271 parameters	Δρ_max_ = 0.28 e Å^−^^3^
0 restraints	Δρ_min_ = −0.41 e Å^−^^3^

### Special details

**Table:**

Geometry. All esds (except the esd in the dihedral angle between two l.s. planes) are estimated using the full covariance matrix. The cell esds are taken into account individually in the estimation of esds in distances, angles and torsion angles; correlations between esds in cell parameters are only used when they are defined by crystal symmetry. An approximate (isotropic) treatment of cell esds is used for estimating esds involving l.s. planes.
Refinement. Refinement of F^2^ against ALL reflections. The weighted R-factor wR and goodness of fit S are based on F^2^, conventional R-factors R are based on F, with F set to zero for negative F^2^. The threshold expression of F^2^ > 2sigma(F^2^) is used only for calculating R-factors(gt) etc. and is not relevant to the choice of reflections for refinement. R-factors based on F^2^ are statistically about twice as large as those based on F, and R- factors based on ALL data will be even larger.

### Fractional atomic coordinates and isotropic or equivalent isotropic displacement parameters (Å^2^)

**Table:**

	*x*	*y*	*z*	*U*_iso_*/*U*_eq_	
C1	0.98198 (5)	0.1534 (5)	0.63987 (11)	0.0788 (6)	
H1A	0.9788	0.3107	0.6558	0.095*	
H1B	0.9869	0.0376	0.6808	0.095*	
C2	0.99781 (5)	0.1272 (5)	0.58082 (13)	0.0856 (7)	
H2A	1.0125	−0.0047	0.5853	0.103*	
H2B	1.0044	0.2683	0.5604	0.103*	
C3	0.96139 (4)	0.0774 (4)	0.55940 (10)	0.0638 (4)	
H3	0.9545	−0.0866	0.5525	0.077*	
C4	0.93845 (4)	0.2511 (3)	0.51216 (9)	0.0519 (4)	
C5	0.90431 (4)	0.1631 (3)	0.46517 (8)	0.0431 (3)	
H5	0.9066	0.0034	0.4482	0.052*	
C6	0.88405 (4)	0.1533 (3)	0.51978 (8)	0.0438 (3)	
C7	0.88474 (4)	0.3324 (3)	0.57192 (8)	0.0528 (4)	
H7	0.8982	0.4612	0.5744	0.063*	
C8	0.86572 (5)	0.3224 (3)	0.62019 (10)	0.0614 (4)	
H8	0.8667	0.4431	0.6549	0.074*	
C9	0.84548 (5)	0.1340 (3)	0.61672 (10)	0.0637 (5)	
H9	0.8328	0.1277	0.6492	0.076*	
C10	0.84387 (5)	−0.0456 (3)	0.56527 (10)	0.0596 (4)	
H10	0.8301	−0.1729	0.5622	0.071*	
C11	0.86323 (4)	−0.0313 (3)	0.51858 (9)	0.0495 (3)	
C12	0.88775 (4)	0.3132 (3)	0.39218 (8)	0.0436 (3)	
H12	0.8869	0.4750	0.4088	0.052*	
C13	0.90741 (3)	0.3082 (2)	0.33675 (7)	0.0416 (3)	
C14	0.92695 (4)	0.4952 (3)	0.33202 (10)	0.0569 (4)	
H14	0.9284	0.6236	0.3641	0.068*	
C15	0.94436 (5)	0.4954 (3)	0.28052 (12)	0.0671 (5)	
H15	0.9573	0.6232	0.2783	0.080*	
C16	0.94255 (4)	0.3079 (3)	0.23278 (11)	0.0645 (5)	
H16	0.9543	0.3078	0.1982	0.077*	
C17	0.92334 (5)	0.1211 (3)	0.23624 (10)	0.0638 (4)	
H17	0.9219	−0.0060	0.2036	0.077*	
C18	0.90601 (4)	0.1203 (3)	0.28822 (9)	0.0537 (4)	
H18	0.8932	−0.0088	0.2905	0.064*	
C19	0.85253 (3)	0.2291 (3)	0.35328 (9)	0.0491 (3)	
H19A	0.8532	0.0695	0.3360	0.059*	
H19B	0.8413	0.2278	0.3915	0.059*	
C20	0.83275 (4)	0.3740 (3)	0.28526 (8)	0.0457 (3)	
C21	0.80262 (3)	0.2639 (3)	0.22939 (8)	0.0436 (3)	
C22	0.79031 (4)	0.0502 (3)	0.24330 (10)	0.0511 (4)	
H22	0.8010	−0.0303	0.2888	0.061*	
C23	0.76239 (4)	−0.0443 (3)	0.19061 (10)	0.0570 (4)	
H23	0.7542	−0.1870	0.2005	0.068*	
C24	0.74683 (4)	0.0746 (3)	0.12351 (10)	0.0596 (4)	
C25	0.75831 (5)	0.2881 (3)	0.10805 (10)	0.0640 (4)	
H25	0.7474	0.3677	0.0625	0.077*	
C26	0.78613 (4)	0.3814 (3)	0.16101 (9)	0.0542 (4)	
H26	0.7940	0.5249	0.1510	0.065*	
O1	0.94554 (4)	0.4569 (2)	0.51323 (8)	0.0786 (4)	
O2	0.84006 (3)	0.5733 (2)	0.27563 (7)	0.0614 (3)	
F1	0.86105 (3)	−0.20686 (17)	0.46719 (6)	0.0687 (3)	
Cl1	0.712537 (14)	−0.05059 (13)	0.05552 (4)	0.0982 (2)	

### Atomic displacement parameters (Å^2^)

**Table:**

	*U*^11^	*U*^22^	*U*^33^	*U*^12^	*U*^13^	*U*^23^
C1	0.0636 (11)	0.1197 (18)	0.0452 (9)	−0.0021 (11)	0.0071 (8)	−0.0002 (10)
C2	0.0520 (10)	0.131 (2)	0.0690 (12)	0.0048 (11)	0.0131 (9)	−0.0203 (13)
C3	0.0525 (9)	0.0754 (12)	0.0553 (9)	−0.0003 (8)	0.0063 (8)	−0.0035 (9)
C4	0.0535 (8)	0.0638 (10)	0.0385 (7)	−0.0064 (7)	0.0153 (6)	−0.0048 (7)
C5	0.0491 (7)	0.0456 (7)	0.0346 (6)	−0.0001 (6)	0.0137 (6)	−0.0022 (5)
C6	0.0514 (8)	0.0468 (7)	0.0332 (6)	0.0044 (6)	0.0139 (6)	0.0040 (5)
C7	0.0671 (9)	0.0514 (8)	0.0399 (7)	0.0029 (7)	0.0177 (7)	−0.0003 (6)
C8	0.0798 (11)	0.0640 (10)	0.0449 (8)	0.0165 (9)	0.0266 (8)	0.0009 (7)
C9	0.0740 (11)	0.0741 (11)	0.0540 (9)	0.0199 (9)	0.0361 (9)	0.0158 (8)
C10	0.0645 (10)	0.0596 (10)	0.0608 (10)	0.0028 (8)	0.0290 (8)	0.0130 (8)
C11	0.0602 (9)	0.0465 (8)	0.0435 (8)	0.0038 (7)	0.0191 (7)	0.0019 (6)
C12	0.0498 (7)	0.0440 (7)	0.0381 (7)	0.0003 (6)	0.0158 (6)	0.0002 (6)
C13	0.0437 (7)	0.0447 (7)	0.0350 (6)	−0.0019 (6)	0.0111 (5)	0.0005 (5)
C14	0.0669 (10)	0.0517 (9)	0.0554 (9)	−0.0145 (8)	0.0247 (8)	−0.0084 (7)
C15	0.0688 (11)	0.0698 (11)	0.0700 (11)	−0.0222 (9)	0.0328 (9)	−0.0019 (9)
C16	0.0640 (10)	0.0797 (12)	0.0605 (10)	−0.0017 (9)	0.0350 (9)	0.0027 (9)
C17	0.0783 (11)	0.0650 (10)	0.0564 (9)	−0.0045 (9)	0.0334 (9)	−0.0131 (8)
C18	0.0648 (9)	0.0505 (8)	0.0509 (8)	−0.0118 (7)	0.0260 (7)	−0.0076 (7)
C19	0.0458 (7)	0.0585 (9)	0.0445 (8)	0.0021 (7)	0.0169 (6)	0.0104 (6)
C20	0.0487 (7)	0.0494 (8)	0.0434 (7)	0.0060 (6)	0.0210 (6)	0.0053 (6)
C21	0.0444 (7)	0.0474 (7)	0.0428 (7)	0.0075 (6)	0.0195 (6)	0.0044 (6)
C22	0.0483 (8)	0.0550 (9)	0.0530 (8)	0.0065 (7)	0.0208 (7)	0.0114 (7)
C23	0.0503 (8)	0.0549 (9)	0.0700 (10)	−0.0021 (7)	0.0253 (8)	0.0060 (8)
C24	0.0470 (8)	0.0689 (11)	0.0603 (10)	−0.0037 (8)	0.0140 (7)	−0.0006 (8)
C25	0.0627 (10)	0.0692 (11)	0.0525 (9)	0.0006 (9)	0.0085 (8)	0.0134 (8)
C26	0.0588 (9)	0.0514 (8)	0.0508 (8)	0.0003 (7)	0.0157 (7)	0.0090 (7)
O1	0.0813 (9)	0.0664 (8)	0.0730 (9)	−0.0223 (7)	0.0049 (7)	−0.0020 (6)
O2	0.0690 (7)	0.0502 (6)	0.0584 (7)	−0.0017 (5)	0.0118 (6)	0.0076 (5)
F1	0.0890 (7)	0.0537 (6)	0.0725 (7)	−0.0151 (5)	0.0387 (6)	−0.0136 (5)
Cl1	0.0709 (3)	0.1139 (5)	0.0894 (4)	−0.0320 (3)	−0.0017 (3)	0.0073 (3)

### Geometric parameters (Å, º)

**Table:**

C1—C2	1.461 (3)	C13—C14	1.382 (2)
C1—C3	1.512 (3)	C13—C18	1.383 (2)
C1—H1A	0.9700	C14—C15	1.382 (2)
C1—H1B	0.9700	C14—H14	0.9300
C2—C3	1.514 (3)	C15—C16	1.369 (3)
C2—H2A	0.9700	C15—H15	0.9300
C2—H2B	0.9700	C16—C17	1.366 (3)
C3—C4	1.467 (3)	C16—H16	0.9300
C3—H3	0.9800	C17—C18	1.387 (2)
C4—O1	1.216 (2)	C17—H17	0.9300
C4—C5	1.525 (2)	C18—H18	0.9300
C5—C6	1.5266 (19)	C19—C20	1.506 (2)
C5—C12	1.551 (2)	C19—H19A	0.9700
C5—H5	0.9800	C19—H19B	0.9700
C6—C11	1.381 (2)	C20—O2	1.2117 (19)
C6—C7	1.394 (2)	C20—C21	1.502 (2)
C7—C8	1.388 (2)	C21—C22	1.389 (2)
C7—H7	0.9300	C21—C26	1.392 (2)
C8—C9	1.375 (3)	C22—C23	1.381 (2)
C8—H8	0.9300	C22—H22	0.9300
C9—C10	1.380 (3)	C23—C24	1.371 (3)
C9—H9	0.9300	C23—H23	0.9300
C10—C11	1.377 (2)	C24—C25	1.382 (3)
C10—H10	0.9300	C24—Cl1	1.7425 (18)
C11—F1	1.3583 (18)	C25—C26	1.378 (2)
C12—C13	1.5182 (19)	C25—H25	0.9300
C12—C19	1.527 (2)	C26—H26	0.9300
C12—H12	0.9800		
			
C2—C1—C3	61.19 (13)	C13—C12—H12	108.1
C2—C1—H1A	117.6	C19—C12—H12	108.1
C3—C1—H1A	117.6	C5—C12—H12	108.1
C2—C1—H1B	117.6	C14—C13—C18	117.53 (13)
C3—C1—H1B	117.6	C14—C13—C12	120.74 (13)
H1A—C1—H1B	114.8	C18—C13—C12	121.72 (13)
C1—C2—C3	61.07 (13)	C13—C14—C15	121.34 (15)
C1—C2—H2A	117.7	C13—C14—H14	119.3
C3—C2—H2A	117.7	C15—C14—H14	119.3
C1—C2—H2B	117.7	C16—C15—C14	120.23 (16)
C3—C2—H2B	117.7	C16—C15—H15	119.9
H2A—C2—H2B	114.8	C14—C15—H15	119.9
C4—C3—C1	116.42 (17)	C17—C16—C15	119.53 (15)
C4—C3—C2	117.72 (19)	C17—C16—H16	120.2
C1—C3—C2	57.75 (13)	C15—C16—H16	120.2
C4—C3—H3	117.2	C16—C17—C18	120.28 (16)
C1—C3—H3	117.2	C16—C17—H17	119.9
C2—C3—H3	117.2	C18—C17—H17	119.9
O1—C4—C3	122.04 (16)	C13—C18—C17	121.08 (15)
O1—C4—C5	121.34 (15)	C13—C18—H18	119.5
C3—C4—C5	116.51 (15)	C17—C18—H18	119.5
C4—C5—C6	107.17 (11)	C20—C19—C12	114.70 (12)
C4—C5—C12	112.84 (12)	C20—C19—H19A	108.6
C6—C5—C12	112.52 (12)	C12—C19—H19A	108.6
C4—C5—H5	108.0	C20—C19—H19B	108.6
C6—C5—H5	108.0	C12—C19—H19B	108.6
C12—C5—H5	108.0	H19A—C19—H19B	107.6
C11—C6—C7	116.25 (13)	O2—C20—C21	120.28 (13)
C11—C6—C5	121.61 (13)	O2—C20—C19	122.37 (14)
C7—C6—C5	122.11 (13)	C21—C20—C19	117.33 (13)
C8—C7—C6	121.30 (16)	C22—C21—C26	118.49 (14)
C8—C7—H7	119.3	C22—C21—C20	122.78 (13)
C6—C7—H7	119.3	C26—C21—C20	118.73 (13)
C9—C8—C7	120.02 (16)	C23—C22—C21	120.96 (15)
C9—C8—H8	120.0	C23—C22—H22	119.5
C7—C8—H8	120.0	C21—C22—H22	119.5
C8—C9—C10	120.37 (15)	C24—C23—C22	119.22 (16)
C8—C9—H9	119.8	C24—C23—H23	120.4
C10—C9—H9	119.8	C22—C23—H23	120.4
C11—C10—C9	118.19 (16)	C23—C24—C25	121.31 (16)
C11—C10—H10	120.9	C23—C24—Cl1	119.07 (14)
C9—C10—H10	120.9	C25—C24—Cl1	119.60 (14)
F1—C11—C10	117.77 (15)	C26—C25—C24	119.07 (16)
F1—C11—C6	118.35 (13)	C26—C25—H25	120.5
C10—C11—C6	123.86 (15)	C24—C25—H25	120.5
C13—C12—C19	111.63 (11)	C25—C26—C21	120.95 (15)
C13—C12—C5	111.38 (12)	C25—C26—H26	119.5
C19—C12—C5	109.43 (12)	C21—C26—H26	119.5
			
C2—C1—C3—C4	−107.5 (2)	C5—C12—C13—C14	−102.94 (16)
C1—C2—C3—C4	105.2 (2)	C19—C12—C13—C18	−44.28 (19)
C1—C3—C4—O1	38.4 (2)	C5—C12—C13—C18	78.37 (17)
C2—C3—C4—O1	−27.3 (3)	C18—C13—C14—C15	0.3 (3)
C1—C3—C4—C5	−137.90 (16)	C12—C13—C14—C15	−178.45 (16)
C2—C3—C4—C5	156.43 (15)	C13—C14—C15—C16	−0.1 (3)
O1—C4—C5—C6	−94.60 (18)	C14—C15—C16—C17	0.2 (3)
C3—C4—C5—C6	81.72 (16)	C15—C16—C17—C18	−0.6 (3)
O1—C4—C5—C12	29.8 (2)	C14—C13—C18—C17	−0.7 (3)
C3—C4—C5—C12	−153.86 (13)	C12—C13—C18—C17	178.06 (15)
C4—C5—C6—C11	−138.34 (15)	C16—C17—C18—C13	0.8 (3)
C12—C5—C6—C11	97.05 (16)	C13—C12—C19—C20	−61.19 (17)
C4—C5—C6—C7	43.57 (18)	C5—C12—C19—C20	175.04 (12)
C12—C5—C6—C7	−81.04 (17)	C12—C19—C20—O2	−20.5 (2)
C11—C6—C7—C8	0.7 (2)	C12—C19—C20—C21	160.82 (12)
C5—C6—C7—C8	178.87 (14)	O2—C20—C21—C22	−170.23 (14)
C6—C7—C8—C9	−0.5 (3)	C19—C20—C21—C22	8.5 (2)
C7—C8—C9—C10	−0.1 (3)	O2—C20—C21—C26	9.2 (2)
C8—C9—C10—C11	0.6 (3)	C19—C20—C21—C26	−172.05 (13)
C9—C10—C11—F1	−178.87 (15)	C26—C21—C22—C23	0.1 (2)
C9—C10—C11—C6	−0.5 (3)	C20—C21—C22—C23	179.58 (14)
C7—C6—C11—F1	178.22 (13)	C21—C22—C23—C24	0.4 (2)
C5—C6—C11—F1	0.0 (2)	C22—C23—C24—C25	−0.7 (3)
C7—C6—C11—C10	−0.2 (2)	C22—C23—C24—Cl1	177.56 (13)
C5—C6—C11—C10	−178.39 (15)	C23—C24—C25—C26	0.6 (3)
C4—C5—C12—C13	61.90 (15)	Cl1—C24—C25—C26	−177.70 (14)
C6—C5—C12—C13	−176.68 (12)	C24—C25—C26—C21	−0.1 (3)
C4—C5—C12—C19	−174.20 (12)	C22—C21—C26—C25	−0.3 (2)
C6—C5—C12—C19	−52.77 (16)	C20—C21—C26—C25	−179.75 (15)
C19—C12—C13—C14	134.40 (15)		

### Hydrogen-bond geometry (Å, º)

**Table:**

*D*—H···*A*	*D*—H	H···*A*	*D*···*A*	*D*—H···*A*
C12—H12···F1^i^	0.98	2.54	3.433 (2)	151

Symmetry code: (i) *x*, *y*+1, *z*.
